# Double Pneumonia, with Chronic Bronchitis, Cured by Sugar of Lead and Sulphate of Quinine

**Published:** 1865-01

**Authors:** G. C. Paoli

**Affiliations:** Chicago, Ill.


ARTICLE III.

### DOUBLE PNEUMONIA, WITH CHRONIC BRONCHITIS, CURED BY SUGAR OF LEAD AND SULPHATE OF QUININE.

By G. C. PAOLI, M.D., of Chicago, Ill.

Read before the Chicago Medical Society.

It is a well established usage, in all diseases, to individualize, and I think more especially in inflammation of the lungs, as the following history will substantiate:—

Mrs. C., 32 years of age, whom I was called to see, on the 8th of January, had been moving to her residence, a few days previous, and had been exposed to the cold atmosphere, and was suddenly, on the 6th of January, taken with a chill; also previous to her sudden illness, she had, for several months, been suffering with dyspnœa, when she exerted herself in going up stairs. When I called to see her, late in the night, I found her very weak, short breathed, with an expression of great anxiety, respiration 60, pulse 120, diarrhœa of several days standing, expectoration frothy mucus, sanguinolant appearance; the tongue nearly natural; pain in the cardiac region on pressure; percussion could only, with very great difficulty, be instituted, as the patient was very weak; the anterior, as well as the posterior, parts dull; crepitation audible, on the whole anterior as well as the posterior part; and at the inferior angle of the scapula, bronchous respiration. In the night, I prescribed vinum antimonii, combined with mucilages, to prevent the increase of the diarrhœa. Next morning, when I called early, I found the patient in a hopeless condition—I can say nearly dying. The extremities cold and bluish, cold sweat over the whole body, a burning thirst, the tongue dry, the pulse small and weak, about 120, respiration hurried, accompanied by mucous rale in trachea, expectoration frothy and rusty.

Although the treatment in this case seemed to me in vain, I still felt that something ought to be done, and I concluded that sugar of lead in this case would be indicated, namely, to diminish somewhat the profuse cold sweat and diarrhœa. To support the strength of the patient, I added sulphate of quinine, namely, quinine, gr. 1, acetas of lead, gr. ½, one powder every other hour.

*Jan.* 10.—The patient somewhat better, collapse less, the pulse 90, respiration 42, the cold sweat less, but the mucous rale of trachea was still present, the tongue dry and covered, diarrhœa not present during the night.

*Jan.* 11.—Respiration 44, pulse 104, the cold sweat had disappeared, the tongue humid, clean, desire for food. The patient had rested during the night; the bowels natural; I did not examine her lungs, because she was feeling so weak.

*Jan.* 12.—Respiration 38, pulse 100, the cough somewhat troublesome, the expectoration purulent, bronchous respiration from the left spina scapula drawn to the base of the lung, and in the right regio-scapularis; on the right anterior surface sonorous rale; hepatization of the lower left and middle lobe of the lung. In order to avoid tediousness in repeating the different symptoms, I will only say, in conclusion, that the patient regained her strength slowly; and that on the 20th, she began to sit up in her bed. It was in this case that I, for the first time, made a chemical examination of the urine. By applying the test, I discovered the absence of chlorides in the first eight days, and on the ninth, the chlorides appeared.

When I take into consideration my patient's critical condition, I must confess, that in this case, sugar of lead saved the patient's life.

It is often very difficult for medical practitioners to give a certain prognosis in acute inflammations of the lungs, and equally difficult to follow certain rules, which are laid down for the treatment of this disease, because the patient's age, constitution, and complications embarrass us more or less in treatment of inflammation of the lungs.

